# Inhibition of GSDMD activation by Z-LLSD-FMK or Z-YVAD-FMK reduces vascular inflammation and atherosclerotic lesion development in ApoE^−/−^ mice

**DOI:** 10.3389/fphar.2023.1184588

**Published:** 2023-08-01

**Authors:** Bao-Li Zhang, Peng Yu, En-Yong Su, Chun-Yu Zhang, Shi-Yao Xie, Xue Yang, Yun-Zeng Zou, Ming Liu, Hong Jiang

**Affiliations:** ^1^ Department of Cardiology, Shanghai Institute of Cardiovascular Diseases, National Clinical Research Center for Interventional Medicine, Zhongshan Hospital, Fudan University, Shanghai, China; ^2^ Department of Endocrinology and Metabolism, Fudan Institute of Metabolic Diseases, Zhongshan Hospital, Fudan University, Shanghai, China; ^3^ Department of Health Management Center, Zhongshan Hospital, Fudan University, Shanghai, China; ^4^ Shanghai Engineering Research Center of AI Technology for Cardiopulmonary Diseases, Zhongshan Hospital, Fudan University, Shanghai, China

**Keywords:** atherosclerosis, GSDMD, macrophage, pyroptosis, vascular inflammation, Z-LLSD-FMK, Z-YVAD-FMK

## Abstract

Pyroptosis is a form of pro-inflammatory cell death that can be mediated by gasdermin D (GSDMD) activation induced by inflammatory caspases such as caspase-1. Emerging evidence suggests that targeting GSDMD activation or pyroptosis may facilitate the reduction of vascular inflammation and atherosclerotic lesion development. The current study investigated the therapeutic effects of inhibition of GSDMD activation by the novel GSDMD inhibitor N-Benzyloxycarbonyl-Leu-Leu-Ser-Asp(OMe)-fluoromethylketone (Z-LLSD-FMK), the specific caspase-1 inhibitor N-Benzyloxycarbonyl-Tyr-Val-Ala-Asp(OMe)-fluoromethylketone (Z-YVAD-FMK), and a combination of both on atherosclerosis in ApoE^−/−^ mice fed a western diet at 5 weeks of age, and further determined the efficacy of these polypeptide inhibitors in bone marrow-derived macrophages (BMDMs). *In vivo* studies there was plaque formation, GSDMD activation, and caspase-1 activation in aortas, which increased gradually from 6 to 18 weeks of age, and increased markedly at 14 and 18 weeks of age. ApoE^−/−^ mice were administered Z-LLSD-FMK (200 µg/day), Z-YVAD-FMK (200 µg/day), a combination of both, or vehicle control intraperitoneally from 14 to 18 weeks of age. Treatment significantly reduced lesion formation, macrophage infiltration in lesions, protein levels of vascular cell adhesion molecule-1 and monocyte chemoattractant protein-1, and pyroptosis-related proteins such as activated caspase-1, activated GSDMD, cleaved interleukin(IL)-1β, and high mobility group box 1 in aortas. No overt differences in plasma lipid contents were detected. *In vitro* treatment with these polypeptide inhibitors dramatically decreased the percentage of propidium iodide-positive BMDMs, the release of lactate dehydrogenase and IL-1β, and protein levels of pyroptosis-related proteins both in supernatants and cell lysates elevated by lipopolysaccharide + nigericin. Notably however, there were no significant differences in the above-mentioned results between the Z-LLSD-FMK group and the Z-YVAD-FMK group, and the combination of both did not yield enhanced effects. These findings indicate that suppression of GSDMD activation by Z-LLSD-FMK or Z-YVAD-FMK reduces vascular inflammation and lesion development in ApoE^−/−^ mice.

## 1 Introduction

Atherosclerosis is a chronic vascular inflammatory disorder in which lesions are formed in the arterial wall. With the progression of lesions, life-threatening manifestations of atherosclerotic cardiovascular diseases (ASCVDs) occur (Ahmadi et al., 2019). Currently the high mortality related to ASCVDs exceeds that attributable to cancer as a primary cause of death globally (Arai et al., 2019; Ma et al., 2020; Virani et al., 2020). Reducing lesion development would reportedly avoid the later stages, and thus prevent clinical manifestations and death. It is therefore of great importance to develop novel pharmacological therapeutic strategies to inhibit atherosclerotic lesion development.

It is well established that vascular inflammation makes a significant contribution to lesion development. It was recently demonstrated that pyroptosis is a pro-inflammatory form of programmed cell death, and gasdermin D (GSDMD), a pore-forming protein, is a final executor of pyroptosis (Shi et al., 2017). GSDMD is cleaved by caspase-1 activated by assembly of diverse canonical inflammasomes, like nucleotide-binding oligomerization domain-like receptor protein 3 (NLRP3), or by caspase-11 (or human caspases 4 and 5) activated by non-canonical inflammasome pathway, into N-terminal GSDMD fragments (GSDMD-N) (Shi J. et al., 2015). GSDMD-N oligomerizes to form pores in the cell membrane, resulting in the release of pro-inflammatory mediators such as cleaved interleukin(IL)-1β (He et al., 2015; Evavold et al., 2018) and high mobility group box 1 (HMGB1) (Volchuk et al., 2020), triggering a strong pro-inflammatory response (Broz et al., 2020). Deficiency of GSDMD in a low-density lipoprotein receptor (LDLr) antisense oligonucleotide-induced hyperlipidemic mouse model reportedly reduced inflammatory responses in the artery wall and limited lesion development (Opoku et al., 2021). The same effects are evidently induced by a lack of caspase-1 (Gage et al., 2012; Usui et al., 2012; Yin et al., 2015) or IL-1β (Kirii et al., 2003), and neutralization or inhibition of HMGB1 (Kanellakis et al., 2011; Liu et al., 2013) in ApoE^−/−^ mice. Macrophages are the major immune cell population in atherosclerotic lesions (Xu et al., 2019), and play a central role in lesion development (Barrett, 2020; Willemsen and de Winther, 2020). Critically, in previous studies pyroptosis-related proteins such as caspase-1, IL-1β were mainly expressed in macrophages in carotid atherosclerotic plaques (Shi X. et al., 2015). Moreover, a previous study has indicated that reconstitution of LDLr^−/−^ mice with bone marrow lacking IL-1α/IL-1β can lead to decreased vascular inflammation and lesion sizes (Duewell et al., 2010). Hence pyroptosis—at least macrophage pyroptosis—plays an essential role in vascular inflammation and lesion development, and suppression of GSDMD activation or cleavage may be a promising strategy for reducing lesion development.

GSDMD binds directly to caspases 1, 4, 5, and 11 (Yang et al., 2018), and is specifically cleaved by them (Shi J. et al., 2015). A specific cleavage site peptide of human GSDMD is _272_FLTD_275_ (Shi J. et al., 2015), and recently N-acetyl-Phe-Leu-Thr-Asp-chloromethylketone (Ac-FLTD-CMK), the human GSDMD inhibitor, has been designed and demonstrated to significantly block GSDMD cleavage and pyroptosis by potently inhibiting the enzymatic activities of caspases 1, 4, 5, and 11 (Yang et al., 2018). Its availability and specificity have also been demonstrated in a series of pharmacological and cellular assays (Yang et al., 2018). To date the therapeutic effects of Ac-FLTD-CMK on atherosclerosis have not been investigated. The specific cleavage site peptide of mouse GSDMD is _273_LLSD_276_ (Shi J. et al., 2015), and based on above-mentioned studies, in the current study N-Benzyloxycarbonyl-Leu-Leu-Ser-Asp(OMe)-fluoromethylketone (Z-LLSD-FMK), a novel mouse GSDMD inhibitor, was chemically designed to suppress mouse GSDMD cleavage. It has been reported that N-Benzyloxycarbonyl-Tyr-Val-Ala-Asp(OMe)-fluoromethylketone (Z-YVAD-FMK) (Garcia-Calvo et al., 1998), a specific caspase-1 inhibitor, inhibited cleavage of GSDMD in HK-2 cells (Wen et al., 2022), reduced kidney injury in brain-dead rats (Liu et al., 2021), and mitigated lung injury caused by PM2.5-induced lung inflammation in mice by impeding pyroptosis (Li et al., 2021). However, the therapeutic effects of Z-YVAD-FMK on atherosclerosis remain unclear.

The aim of the present study was to investigate the therapeutic effects of inhibition of GSDMD activation by Z-LLSD-FMK, Z-YVAD-FMK, and the combination of both on vascular inflammation and atherosclerosis. Using *in vivo* and *in vitro* experiments, both Z-LLSD-FMK and Z-YVAD-FMK were found to observably inhibit GSDMD activation and pyroptosis, and reduce vascular inflammation and the lesion development, with no differences or synergies in their effects.

## 2 Materials and methods

### 2.1 Reagents

Z-LLSD-FMK (purity >98%) and Z-YVAD-FMK (purity >98%) were synthesized by GL Biochem Co., Ltd. (Shanghai, China). Dimethylsulfoxide (DMSO), hematoxylin and eosin (H&E), oil red O, lipopolysaccharide (LPS), and nigericin were purchased from Sigma-Aldrich (St. Louis, MO, United States). Paraformaldehyde (PFA) was purchased from Solarbio (Beijing, China). Phosphate-buffered saline (PBS) was purchased from HyClone (Logan, UT, United States). Bovine serum albumin (BSA) was purchased from Yeasen Biotechnology Co., Ltd. (Shanghai, China). Antifade mounting medium with 4′-6-diamidino-2-phenylindole (DAPI), propidium iodide (PI)/Hoechst 33342, and lactate dehydrogenase (LDH) cytotoxicity assay kits were purchased from Beyotime Biotechnology (Shanghai, China). Red blood cell lysis buffer was purchased from Biolegend (San Diego, CA, United States). RPMI 1640 was purchased from Keygen BioTECH (Nanjing, China). Fetal bovine serum (FBS) was purchased from ThermoFisher Scientific (Waltham, MA, United States), and macrophage colony-stimulating factor (M-CSF) was purchased from Peprotech (Rocky Hill, NJ, United States). Mouse IL-1β enzyme-linked immunosorbent assay (ELISA) kits were purchased from R&D systems (Minneapolis, MN, United States). Phosphatase and protease antagonist cocktails were purchased from Sangon Biotech Co., Ltd. (Shanghai, China). Primary antibodies against EGF-like module-containing mucin-like hormone receptor-like 1 (F4/80^+^), HMGB1, caspase-1, GSDMD, and goat anti-rat IgG conjugated to Alexa Fluor 568 were purchased from Abcam (Cambridge, United Kingdom). Primary antibodies against NLRP3 were purchased from Cell Signaling Technology (Beverly, MA, United States), and primary antibodies against vascular cell adhesion molecule-1 (VCAM-1), monocyte chemoattractant protein-1 (MCP-1), and IL-1β were purchased from Wuhan Boster Bioengineering Ltd. (Wuhan, China). Horseradish peroxidase (HRP)-conjugated rabbit secondary IgG was purchased from Jackson ImmunoResearch Laboratories, Inc. (Philadelphia, PA, United States). RIPA lysis buffer, BCA protein assay kits, and enhanced chemiluminescent solutions were purchased from Pierce Biotechnology (Rockford, IL, United States). HRP-conjugated antibody against actin was purchased from Bioworld Technology, Inc. (Bloomington, MN, United States).

### 2.2 Mice and diets

ApoE^−/−^ mice (male, C57BL/6 background, 5 weeks old) were provided by Shanghai Model Organisms Center, Inc. (Shanghai, China) and kept in microisolator cages with water and food available *ad libitum*, under specific pathogen-free conditions at the Shanghai Model Organisms Center. ApoE^−/−^ mice were fed a western diet (21% fat, 0.15% cholesterol; SLAC, Shanghai, China) (Liu et al., 2013) from 5 weeks of age, and samples were collected at 6, 8, 11, 14, and 18 weeks of age (5 mice per group) to determine the timepoint and duration of pharmacological intervention. Thirty-six 5-week-old ApoE^−/−^ mice were also fed the western diet, and at 14 weeks of age these mice were randomized into four groups. Treatments were administered to the four groups via intraperitoneal injection for 4 weeks, as follows: 1) control (vehicle, 0.25 mL, 2% DMSO/day), 2) Z-YVAD-FMK (Fu et al., 2019) (0.25 mL, 200 μg/day), 3) Z-LLSD-FMK (0.25 mL, 200 μg/day), and 4) Z-YVAD-FMK + Z-LLSD-FMK (0.25 mL, 200 μg + 200 μg/day). Mice were killed at 18 weeks of age. All mouse studies were approved by the Animal Ethics Committee of Zhongshan Hospital, Fudan University. All experimental procedures were performed in accordance with the guidelines for the Care and Use of Laboratory Animals published by the US National Institutes of Health (NIH Publication No. 85-23, revised 1996) (Zhang et al., 2021).

### 2.3 Lipid measurement

Blood was collected from the right ventricle of fasted mice after anesthesia. Plasma levels of triglycerides, total cholesterol, and high-density lipoprotein (HDL) were detected via colorimetric enzymatic assays (Jinan Sysmex Limited Company, Jinan, China) in accordance with the manufacturer’s instructions (Liu et al., 2017).

### 2.4 Quantification of atherosclerotic lesions

Atherosclerotic lesions were quantified as previously described (Liu et al., 2013; Liu et al., 2017). In brief, ApoE^−/−^ mice were fasted for more than 4 h then anesthetized. Their hearts were removed, and the aortic arches were dissected. The aortic roots were then harvested and briefly fixed in ice-cold 4% PFA for 4 h. The fixed aortic roots were washed in PBS for 15 min three times, dehydrated in 30% sucrose overnight, then embedded in optimal cutting temperature compound (Sakura, Torrance, CA, United States) followed by snap freezing. Serial sections 10 μm thick were cut from the first appearance of the aortic valve leaflets until the leaflets were no longer visible. Six sections were mounted onto each slide for a total of five slides. Sections were stained with H&E and oil red O, and quantification of the atherosclerotic lesion area was measured by a blinded observer using ImageJ v.1.42q software (National Institutes of Health, Bethesda, MD, United States).

### 2.5 Immunofluorescence

The immunofluorescence procedures applied to cryosections have been described previously (Liu et al., 2013; Liu et al., 2017). Briefly, after drying for 60 min, cryostat sections that had been fixed in 4% PFA were rehydrated in PBS, degreased in 95% ethanol for 10 min, and blocked with PBS containing 2% nonimmune serum solution and 5% BSA at room temperature for 30 min. The cryosections were then incubated with rat anti-F4/80^+^ antibody (1:200) diluted in blocking reagent at 4°C overnight. Negative controls were run in parallel with the omission of primary antibodies. Subsequently, for visualization, sections that had been washed three times with PBS were incubated with goat anti-rat IgG conjugated to Alexa Fluor 568 (1:400) diluted in blocking reagent at room temperature for 2 h. After washing three times with PBS, sections were stained with antifade mounting medium with DAPI. Quantitative analysis of the F4/80-positive area in atherosclerotic lesions was conducted with ImageJ v.1.42q software.

### 2.6 BMDM culture and treatment

Murine BMDMs were obtained from 10 to 12-week-old C57BL/6 male mice and differentiated as described previously (Yanai et al., 2013). Briefly, bone marrow cells in bilateral hind femora and tibias from mice were gently flushed out with 20 mL sterile PBS and collected by centrifugation at 1,500 rpm for 5 min at 4°C. After lysing erythrocytes with red blood cell lysis buffer, cells were cultured and differentiated in 6-cm dishes or 24-well plates with RPMI 1640 supplemented with 10% (vol/vol) FBS and M-CSF (100 ng/mL) for 6 days in a humidified incubator with 5% CO_2_ at 37°C. After the medium was replaced with fresh medium without FBS or M-CSF, BMDMs were pretreated with Z-YVAD-FMK (100 µM), Z-LLSD-FMK (100 µM), or Z-YVAD-FMK (100 µM) + Z-LLSD-FMK (100 µM) for 30 min, then primed with LPS (500 ng/mL) for 4 h followed by nigericin (10 μM) for 30 min (Rozman-Pungercar et al., 2003; Gicquel et al., 2015; Zhang et al., 2020). After treatment, supernatants were collected and centrifuged at 1,500 rpm for 5 min at 4°C to generate cell-free medium preparations. The cell-free supernatants and cells in 6-cm dishes or 24-well plates were then prepared to perform a series of assays.

### 2.7 Cell death assay

The percentage of BMDM death was measured via PI/Hoechst 33342 staining. Briefly, after treatment as described above, samples from each group were washed with PBS and stained with PI and Hoechst 33342 at 4°C for 30 min in the dark in accordance with the manufacturer’s instructions. Cell images were visualized at ×100 magnification under a Leica microscope (Wetzlar, Germany) and analyzed using ImageJ v.1.42q software. The percentage of PI-positive cells was determined in five randomly selected image fields for each group.

LDH cytotoxicity assay kits were also used to evaluate cell death. This assay detects leakage of the intracellular enzyme LDH upon cellular injury. Briefly, after treatment, 120 μL of cell-free supernatant collected from each well in each group was transferred to 96-well plates. Then, 60 μL of the LDH cytotoxicity assay kit reagent mixture was added to each well in the 96-well plates and they were incubated for 30 min at room temperature in the dark. The absorbance signal was determined at 490 nm using a microplate reader (BioTek Instrumentals, Inc., Winooski, VT, United States). Results are shown as the percentage of the total amount of LDH. All of these procedures were performed in accordance with the manufacturer’s instructions.

### 2.8 ELISA

ELISA kits were used to measure concentrations of IL-1β in cell-free supernatants from BMDM cultures, in accordance with the manufacturer’s instructions.

### 2.9 Western blotting

As described previously (Liu et al., 2017), total proteins were extracted from aortic tissue and BMDMs in ice-cold RIPA lysis buffer with a phosphatase and protease antagonist cocktail and disruption by an ultrasonic homogenizer. Protein concentrations were detected with a BCA protein assay kit. Equal volumes of cell culture supernatants were concentrated by centrifugation at 4,000 rpm for 40 min at 4°C using an Amicon^®^ Ultra-4 centrifugal filter with a 10-kDa cutoff (Millipore, Burlington, VT, United States). All of these procedures were conducted in accordance with the manufacturer’s instructions. Equivalent quantities of homogenized aortal or cellular proteins or equal volumes of concentrated supernatants were separated on 15% SDS-PAGE gels by electrophoresis then transferred to polyvinylidene fluoride membranes (Millipore). The primary antibodies were directed against HMGB1 (1:3,000), caspase-1 (1:1,000), GSDMD (1:500), NLRP3 (1:1,000), VCAM-1 (1:400), MCP-1 (1:400), and IL-1β (1:400). After blocking with 5% BSA at room temperature for 1.5 h, the membranes were incubated with the above-mentioned primary antibodies at 4°C overnight. On the second day, after washing three times in Tris-buffered saline with Tween 20 buffer the membranes were probed with HRP-conjugated rabbit secondary IgG antibody (1:20,000) at room temperature for 2 h. An enhanced chemiluminescent solution was used to visualize protein expression. Quantitative analysis of band density was conducted with Quantity One v.4.6.2 software (Bio-Rad, Berkeley, CA, United States). The bands were normalized to β-actin (1:20,000).

### 2.10 Statistical analysis

Data are expressed as the mean ± the standard error of the mean (SEM). Statistical comparisons between multiple groups were performed by one-way analysis of variance followed by a *post hoc* Tukey’s test. All statistical analyses and figure editing were conducted with GraphPad Prism v.6.01 software (GraphPad Software, Inc., La Jolla, CA, United States). Differences were considered significant if *p* < 0.05.

## 3 Results

### 3.1 GSDMD activation in atherosclerotic lesion development in ApoE^−/−^ mice

**FIGURE 1:**
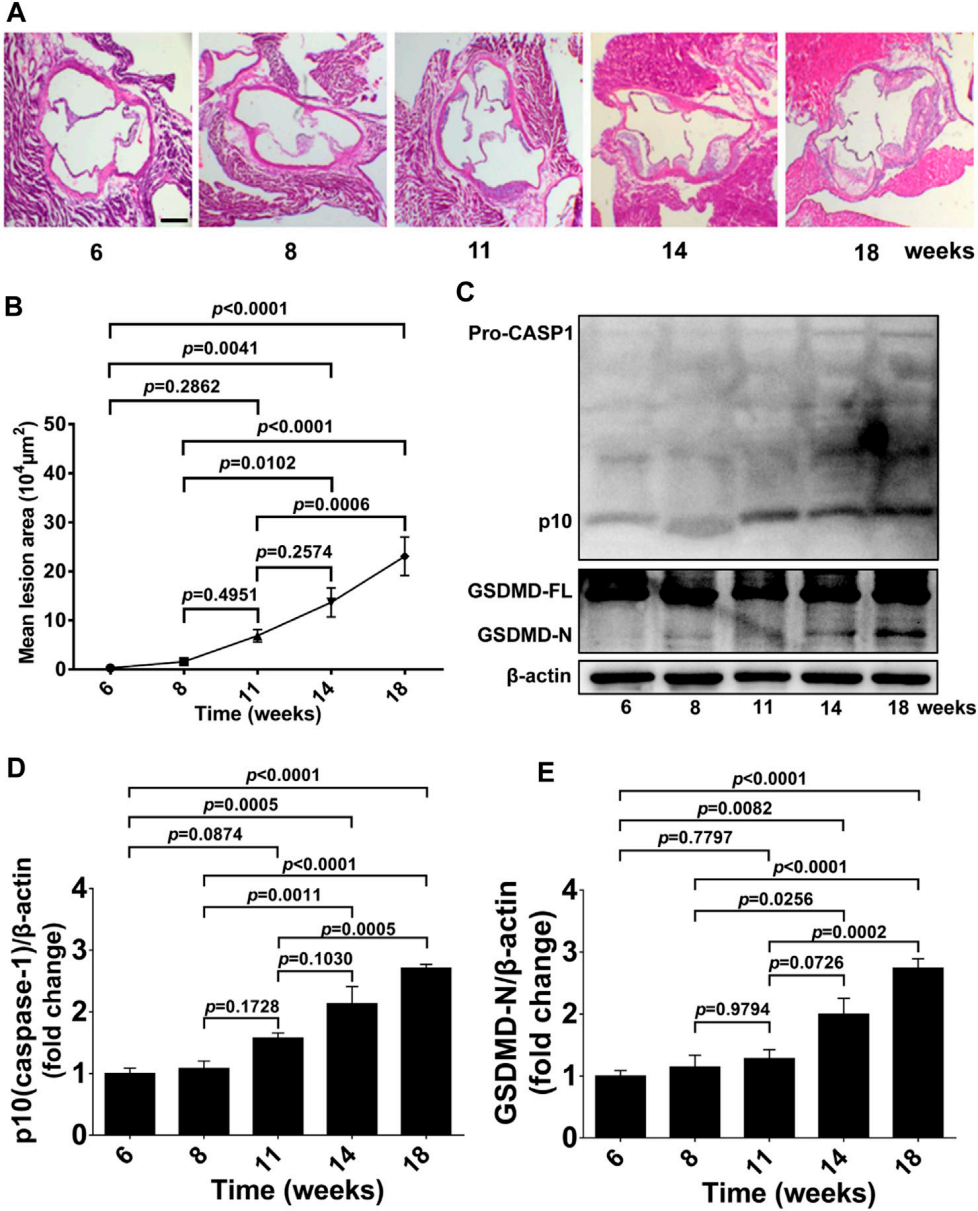
GSDMD activation in atherosclerotic lesion development in ApoE^−/−^ mice. ApoE^−/−^ mice were killed at the ages of 6, 8, 11, 14, and 18 weeks, and their aortas were harvested. **(A)** Representative aortic root cross-sections stained with H&E (scale bar = 200 μm). **(B)** Quantification of lesion area (*n* = 5). **(C)** Representative images of western blots. **(D,E)** Quantification of protein expression of p10 (caspase-1) and GSDMD-N by Western blotting (*n* = 4). β-actin served as the loading control. Data represent means ± SEM.

We first examined atherosclerotic plaque formation in aortic roots, GSDMD activation, and caspase-1 activation in aorta tissue from 6 to 18-week-old ApoE^−/−^ mice fed a western diet to determine the timepoint and duration of pharmacological intervention. Lesion formation in aortic roots was gradually increased from 6 to 18 weeks of age, and significantly increased lesion area was detected in 14 and 18-week-old ApoE^−/−^ mice, whereas no significant plaque formation was evident in mice at 11 weeks of age compared with 6 or 8-week-old ApoE^−/−^ mice (Figures 1A, B). Consistently, protein levels of activated GSDMD (GSDMD-N) and activated caspase-1 (p10) in aortas of ApoE^−/−^ mice were also gradually increased during the development of atherosclerosis, and significant GSDMD activation and caspase-1 activation was observed in the 14 and 18-week-old ApoE^−/−^ mice but not in 11-week-old mice compared with 6 or 8-week-old ApoE^−/−^ mice (Figures 1C–E).

### 3.2 Inhibition of GSDMD activation reduced atherosclerotic lesion development in ApoE^−/−^ mice

**FIGURE 2:**
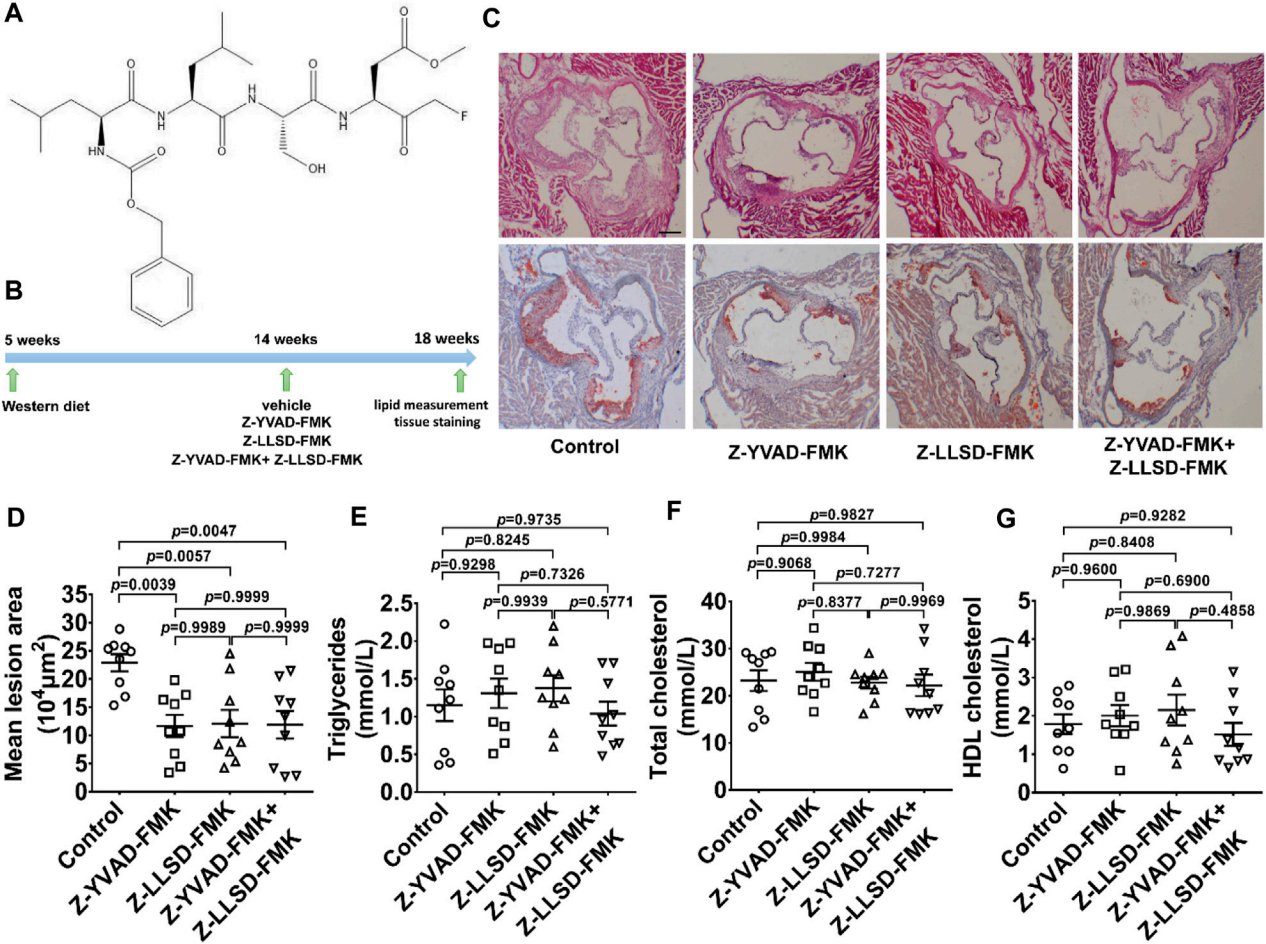
Inhibition of GSDMD activation reduced atherosclerotic lesion development in ApoE^−/−^ mice. After 4 weeks of administration of Z-YVAD-FMK, Z-LLSD-FMK, or the two combined, the ApoE^−/−^ mice were killed at 18 weeks. **(A)** Chemical structure of Z-LLSD-FMK (C_29_H_43_FN_4_O_9_). **(B)** Experimental protocol for the administration of Z-YVAD-FMK, Z-LLSD-FMK, and the two combined in ApoE^−/−^ mice. **(C)** Representative images of aortic sinus sections stained with H&E and oil red O are shown (scale bar = 200 μm). **(D)** Quantitative analysis of atherosclerotic lesion area in the aortic sinus (*n* = 9). Plasma levels of triglycerides **(E)**, total cholesterol **(F)**, and HDL cholesterol **(G)** (*n* = 9).

To determine the therapeutic effects of Z-LLSD-FMK (Figure 2A), Z-YVAD-FMK, and the combination of the two on atherosclerosis, an ApoE^−/−^ mice model of atherosclerosis was used. The above-mentioned agents were administered to ApoE^−/−^ mice at 14 weeks of age, and aortic sinus assays and plasma lipid measurement were performed at 18 weeks of age. The corresponding *in vivo* protocol is presented in Figure 2B. H&E and oil red O staining of aortic root sections revealed that Z-YVAD-FMK, Z-LLSD-FMK, and the two combined markedly ameliorated atherosclerotic plaque development in ApoE^−/−^ mice (Figures 2C, D). Aortic sections exhibited an approximately 49.01% decrease in atherosclerotic lesion area in the Z-YVAD-FMK group, a 47.10% decrease in the Z-LLSD-FMK group, and a 48.04% decrease in the Z-YVAD-FMK + Z-LLSD-FMK group, compared with the control (Figure 2D). Relative to the control group there were no significant differences in triglycerides, total cholesterol or HDL cholesterol in the three intervention groups (Figures 2E–G).

### 3.3 Inhibition of GSDMD activation alleviated vascular inflammation in ApoE^−/−^ mice

**FIGURE 3:**
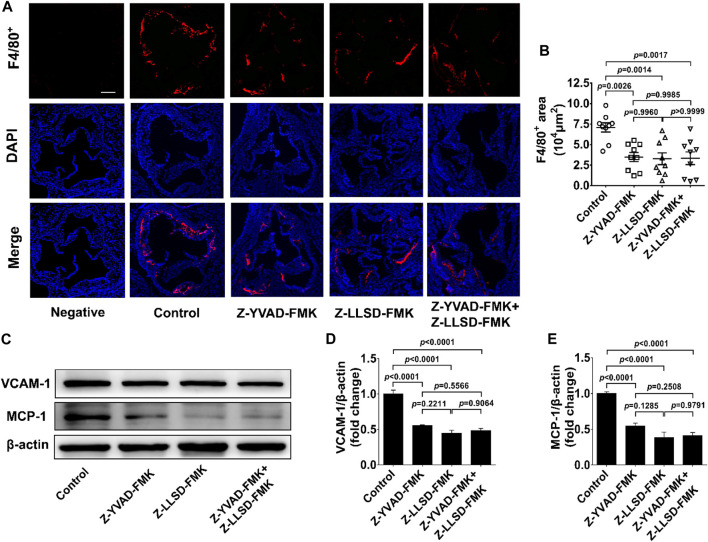
Inhibition of GSDMD activation alleviated vascular inflammation in ApoE^−/−^ mice. The ApoE^−/−^ mice were killed at 18 weeks and aortas were acquired. **(A)** Accumulation of macrophages in atherosclerotic lesions in the aortic sinus was detected by immunofluorescent staining of F4/80^+^ (red) and DAPI (blue), and representative images are shown (scale bar = 200 μm). **(B)** Quantification of the positive area of F4/80^+^ macrophages in atherosclerotic lesions (*n* = 9). **(C)** Western blotting analysis was performed to examine the protein levels of VCAM-1 and MCP-1 in aortas from the indicated mice. **(D,E)** Quantitative analysis (*n* = 6). β-actin served as a loading control. Data represent means ± SEM.

To clarify the inhibitory effects of Z-LLSD-FMK, Z-YVAD-FMK, and the two combined on vascular inflammation, levels of macrophage infiltration and the two pro-inflammatory mediators VCAM-1 and MCP-1 were assessed. After 4 weeks of intervention there was an approximately 50.87% reduction in the positive area of F4/80^+^ macrophages in the atherosclerotic lesions in the aortic sinus in the Z-YVAD-FMK group, a 53.78% reduction in the Z-LLSD-FMK group, and a 52.95% reduction in the Z-YVAD-FMK + Z-LLSD-FMK group, compared with the control group (Figures 3A, B). ApoE^−/−^ mice treated with Z-YVAD-FMK, Z-LLSD-FMK, or the two combined expressed significantly lower protein levels of VCAM-1 and MCP-1 in aorta tissue than vehicle-treated ApoE^−/−^ mice (Figures 3C–E), which corresponded with the decreases in atherosclerotic lesion area and macrophage infiltration (Figure 2D, Figure 3B).

### 3.4 Inhibition of GSDMD activation suppressed expression of pyroptosis pathway-related proteins in ApoE^−/−^ mice

**FIGURE 4:**
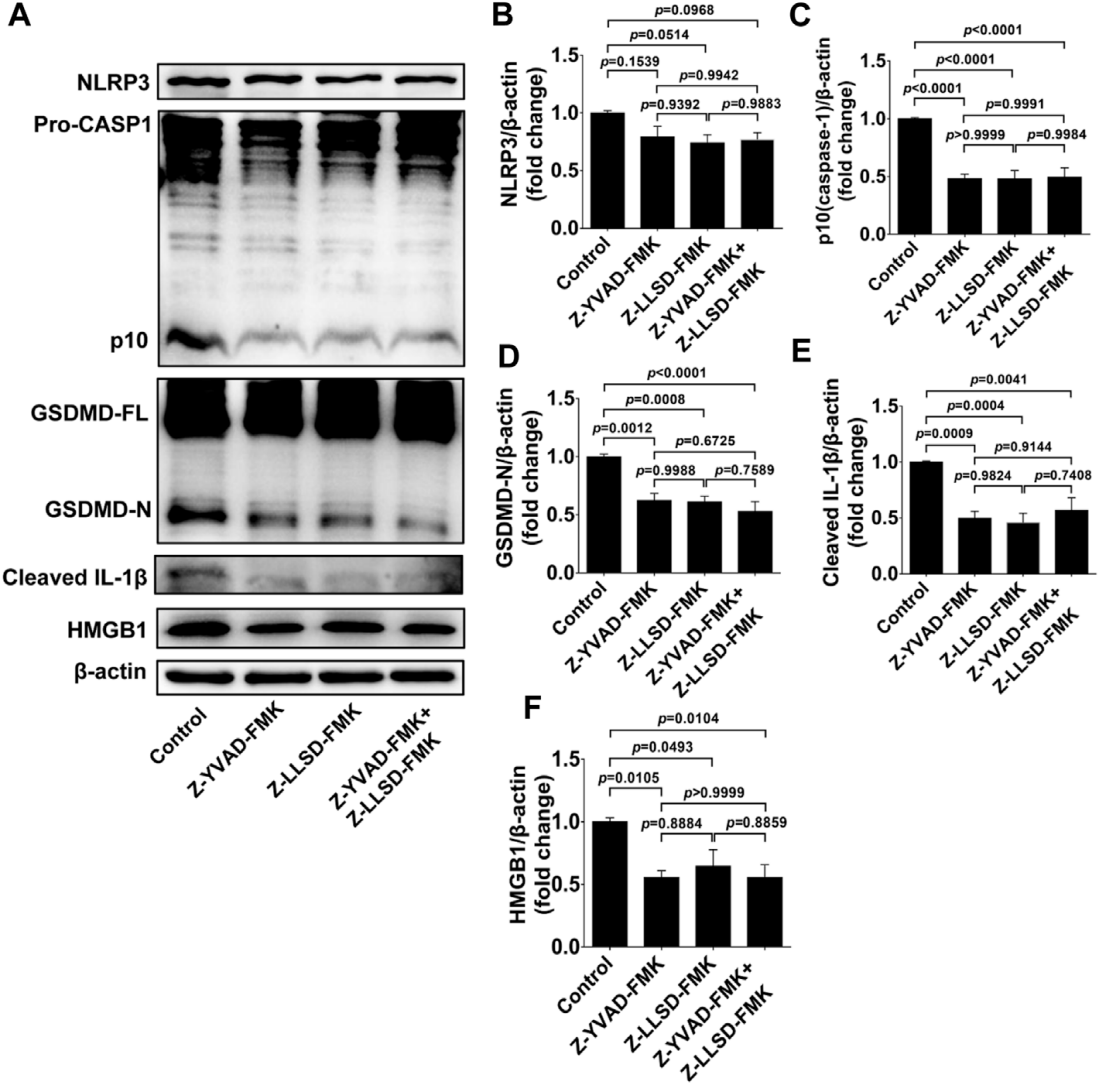
Inhibition of GSDMD activation suppressed the expression of pyroptosis pathway-related proteins in ApoE^−/−^ mice. After 4 weeks of treatment the ApoE^−/−^ mice were killed at 18 weeks. **(A)** Western blotting analysis was conducted to detect protein levels of NLRP3, caspase-1, GSDMD, cleaved IL-1β, and HMGB1 in the aorta. **(B–F)** Quantitative analysis of expression of NLRP3, p10 (caspase-1), GSDMD-N, cleaved IL-1β, and HMGB1 (*n* = 6). β-actin served as the loading control. Data represent means ± SEM.

To confirm the suppressive effects of Z-LLSD-FMK, Z-YVAD-FMK, and the two combined on the expression of pyroptosis pathway-related proteins in ApoE^−/−^ mice, expression of these proteins in whole aortas was assessed in each group. Protein levels of NLRP3 were slightly weakened by Z-YVAD-FMK, Z-LLSD-FMK, and the two combined, but protein levels of activated caspase-1 (p10) and activated GSDMD (GSDMD-N) were remarkably reduced in ApoE^−/−^ mice administered Z-YVAD-FMK, Z-LLSD-FMK, or the two combined, compared with ApoE^−/−^ mice treated with vehicle (Figures 4A–D). Western blotting analysis also showed that the protein levels of cleaved IL-1β and HMGB1 were significantly decreased after administration of Z-YVAD-FMK, Z-LLSD-FMK, or the two combined relative to vehicle treatment (Figures 4A,E,F).

### 3.5 Z-LLSD-FMK or Z-YVAD-FMK restrained GSDMD activation or pyroptosis induced by LPS + nigericin in BMDMs

**FIGURE 5:**
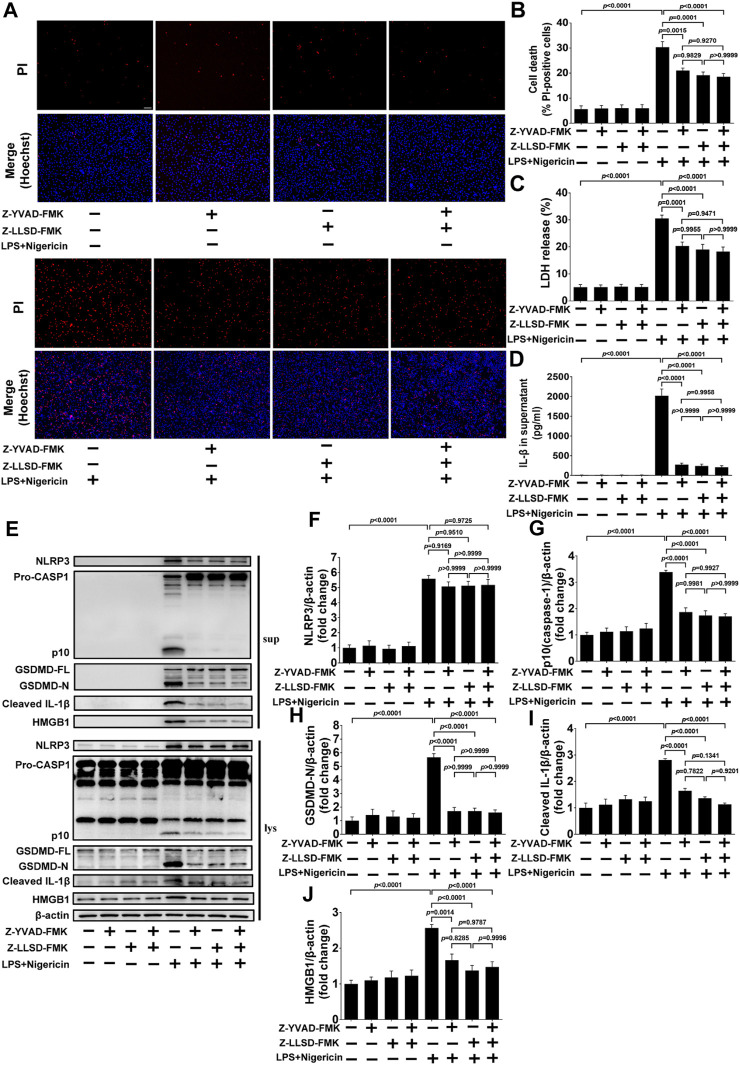
Z-LLSD-FMK or Z-YVAD-FMK inhibited GSDMD activation or pyroptosis induced by LPS + nigericin in BMDMs. BMDMs were primed with LPS for 4 h followed by nigericin for 30 min. Z-LLSD-FMK, Z-YVAD-FMK, and both combined were added 30 min before LPS + nigericin treatment. **(A)** Representative immunofluorescence images of cell death determination via PI (red) and Hoechst 33342 (blue) staining (scale bar = 75 μm). **(B)** The percentage of PI-positive BMDMs was calculated at five randomly selected image fields (*n* = 5). **(C)** LDH release in supernatants (*n* = 5). **(D)** IL-1β release in supernatants was analyzed by ELISA (*n* = 5). **(E)** Western blotting analysis was performed to detect protein levels of NLRP3, caspase-1, GSDMD, cleaved IL-1β, and HMGB1 in supernatants and cell lysates. **(F–J)** Quantitative analysis of expression of NLRP3, p10 (caspase-1), GSDMD-N, cleaved IL-1β, and HMGB1 in cell lysates (*n* = 5). β-actin served as the loading control. Data represent means ± SEM.

To further confirm the inhibitory effects of Z-LLSD-FMK, Z-YVAD-FMK, and the two combined on GSDMD activation or pyroptosis *in vitro*, BMDMs were pretreated with Z-LLSD-FMK, Z-YVAD-FMK, or both for 30 min then primed with LPS for 4 h followed by nigericin for 30 min. Cell death was then determined via staining with PI/Hoechst 33342 and LDH release in supernatants. IL-1β release in supernatants and protein levels of pyroptosis pathway-related proteins both in supernatants and cell lysates were also detected. Compared with the vehicle-treated group, the percentage of PI-positive BMDMs, release of LDH and IL-1β in supernatants, and protein levels of NLRP3, p10 (caspase-1), GSDMD-N, cleaved IL-1β, and HMGB1 in supernatants and cell lysates were significantly elevated in the LPS + nigericin-stimulated group (Figures 5A–J). Remarkably, compared with the control group the percentage of PI-positive BMDMs, release of LDH and IL-1β, protein levels of NLRP3, p10 (caspase-1), GSDMD-N, cleaved IL-1β, and HMGB1 in supernatants, and protein expression of p10 (caspase-1), GSDMD-N, cleaved IL-1β, and HMGB1 in cell lysates were significantly lower in the LPS + nigericin-stimulated groups that were pretreated with Z-LLSD-FMK, Z-YVAD-FMK, or both (Figures 5A–J). However, compared with the control group, protein expression of NLRP3 in cell lysates was slightly decreased in the LPS + nigericin-stimulated groups pretreated with Z-LLSD-FMK, Z-YVAD-FMK, or both (Figures 5E, F).

## 4 Discussion

In the present study inhibition of GSDMD activation by a novel mouse GSDMD inhibitor, Z-LLSD-FMK, and a specific caspase-1 inhibitor, Z-YVAD-FMK, significantly suppressed the expression of pyroptosis pathway-related proteins, and reduced vascular inflammation and lesion development in an ApoE^−/−^ mouse model of atherosclerosis. Both Z-LLSD-FMK and Z-YVAD-FMK markedly inhibited GSDMD activation and pyroptosis in a BMDM model stimulated by LPS + nigericin. There were no significant differences in plasma lipid levels between the control group and treatment groups. Moreover, there were no significant differences in the above-mentioned results between the Z-LLSD-FMK group and the Z-YVAD-FMK group, and the combination of both Z-LLSD-FMK and Z-YVAD-FMK did not have enhanced effects. These results suggest that the canonical inflammasome pathway probably plays a more prominent role in vascular inflammation and lesion development in ApoE^−/−^ mice, and that the novel human GSDMD inhibitor, Ac-FLTD-CMK, may be a novel and effective drug to treat atherosclerosis in the future.

The high mortality related to ASCVDs has been the leading cause of death worldwide (Libby, 2021). Limiting lesion development avoids the later stages of plaques, and thereby prevents clinical manifestations and death (Soehnlein and Libby, 2021). There is thus a great need to investigate potential pharmacological therapeutic strategies to decrease lesion development. Consistent with previous studies (Duewell et al., 2010; Yin et al., 2015; Su et al., 2022), we found that lesion formation in aortic roots and activated caspase-1 in aorta tissue were steadily increased with diet feeding. In the present study, plaque formation, GSDMD activation and caspase-1 activation were gradually increased from 6 to 18 weeks of age. All of them were dramatically increased at 14 and 18 weeks of age, but not at 11 weeks of age compared with 6 or 8 weeks of age. Consequently, we administered Z-LLSD-FMK or Z-YVAD-FMK to ApoE^−/−^ mice at 14 weeks of age, and therapeutic effects on atherosclerosis were detected at 18 weeks of age. Suppression of GSDMD activation by Z-LLSD-FMK or Z-YVAD-FMK remarkably reduced lesion development in ApoE^−/−^ mice, suggesting that inhibition of GSDMD activation may be a promising strategy for the treatment of atherosclerosis.

In previous studies there were no overt differences in plasma levels of triglycerides or total cholesterol between littermate controls and atherosclerosis-prone mice deficient in GSDMD or caspase-1, but macrophage infiltration in lesions and expression of VCAM-1 and IL-1β in aorta tissue were significantly decreased in atherosclerosis-prone mice lacking GSDMD or caspase-1 (Gage et al., 2012; Usui et al., 2012; Yin et al., 2015; Opoku et al., 2021). Likewise, in the current study inhibition of GSDMD activation by Z-LLSD-FMK or Z-YVAD-FMK had the same above-described effects. We also observed that suppression of GSDMD cleavage by Z-LLSD-FMK or Z-YVAD-FMK markedly reduced the protein levels of MCP-1 and HMGB1 in aorta tissue in ApoE^−/−^ mice. Suppression of GSDMD activation by Z-LLSD-FMK or Z-YVAD-FMK attenuated vascular inflammation. It is well-established that imbalanced lipid metabolism and chronic vascular inflammation promote atherosclerosis (Bäck et al., 2019; Libby, 2021). The reduction in atherosclerotic lesion development in the present study was probably due to the relief of vascular inflammation rather than lipid lowering. Previous studies also showed that in spite of optimal low LDL cholesterol concentrations, there is residual risk of ASCVDs during statin therapy (Johannesen et al., 2021). Thus, in addition to lipid-lowering therapy, ameliorating vascular inflammation may also be a crucial strategy for treating atherosclerosis, and suppression of GSDMD activation may be a promising therapeutic strategy.

In the current study inhibition of GSDMD activation by Z-LLSD-FMK or Z-YVAD-FMK significantly reduced macrophage infiltration in lesions. It has previously been reported that a lack of GSDMD in mice or in BMDMs, and deficiency of caspase-1 in ApoE^−/−^ mice, reduced the release or expression of IL-1β (Gage et al., 2012; Usui et al., 2012; Opoku et al., 2021). In other previous studies deficiency of GSDMD in BMDMs, or lack of caspase-1 either in mice or in BMDMs reduced the release of HMGB1 (Lamkanfi et al., 2010; Volchuk et al., 2020). Deficiency of IL-1β in ApoE^−/−^ mice evidently reduced expression of VCAM-1 and MCP-1 (Kirii et al., 2003), whereas exposure of IL-1β caused expression of these molecules in human vascular smooth muscle cells (Wang et al., 1995). Moreover, neutralization or inhibition of HMGB1 in ApoE^−/−^ mice reduced expression of VCAM-1 and MCP-1 (Kanellakis et al., 2011; Liu et al., 2013). Consistent with these studies, we found that suppression of GSDMD activation by Z-LLSD-FMK or Z-YVAD-FMK led to reduced expression or release of IL-1β and HMGB1, which reduced protein levels of both VCAM-1 and MCP-1. This may have led to reduced monocyte adhesion to the arterial wall and reduction in intraplaque macrophages, because MCP-1 is one of the most potent chemoattractants of monocytes, and VCAM-1 plays a critical role in the recruitment of monocytes. In this regard it has been established that inhibition of MCP-1 reduces macrophage levels in atherosclerotic lesions, and blocking VCAM-1 inhibits monocytes from entering the arterial wall (Huo et al., 2000; Inoue et al., 2002).

Previous studies have shown that activation of caspase-1 or the canonical inflammasome pathway is induced by infectious or non-infectious challenges (Shi et al., 2017), and the non-canonical pathway is activated by recognizing cytosolic LPS that originally exists in the cell wall of Gram-negative bacterium (Hagar et al., 2013), thereby inducing GSDMD cleavage or pyroptosis (Shi J. et al., 2015; Kayagaki et al., 2015; Aglietti et al., 2016). In the current study there were no significant differences between the Z-LLSD-FMK group and the Z-YVAD-FMK group in the lesion area, and the combination of both did not have enhanced effects. From our results, the canonical inflammasome pathway probably plays a more prominent role than the non-canonical pathway in promoting vascular inflammation and lesion development in ApoE^−/−^ mice. Maybe it is because atherosclerosis is chronic sterile inflammation, and it has been reported that the effects of the non-canonical pathway are evident in acute infectious inflammation such as *Salmonella* infection (Broz et al., 2012) or LPS-induced sepsis (Napier et al., 2016). However, more further studies need to be undertaken to verify the effects of the non-canonical pathway in atherosclerosis in the future.

It has been verified that the cleavage site peptide of human GSDMD is _272_FLTD_275_, and Ac-FLTD-CMK, the human GSDMD inhibitor, has been designed and confirmed to markedly inhibit GSDMD cleavage in THP-1 cells (Yang et al., 2018). However, the therapeutic effects of Ac-FLTD-CMK on atherosclerosis have not been investigated. In the present study Z-LLSD-FMK, a novel mouse GSDMD inhibitor, was designed because the cleavage site peptide of mouse GSDMD is _273_LLSD_276_, and it has been shown to suppress GSDMD activation *in vivo* and *in vitro*, and more importantly, reduce atherosclerotic lesion development in ApoE^−/−^ mice. GSDMD cleavage leads to production of GSDMD-N that oligomerizes to form pores in membranes (Shi J. et al., 2015), which mediate the release of intracellular pro-inflammatory contents such as IL-1β (He et al., 2015; Evavold et al., 2018). In a previous study inhibition of GSDMD cleavage by Ac-FLTD-CMK reduced IL-1β release *in vitro* (Yang et al., 2018), and in the current study inhibition of GSDMD activation by Z-LLSD-FMK reduced IL-1β expression or release *in vivo* and *in vitro*. In human carotid or coronary atherosclerotic plaques, IL-1β levels were reportedly increased compared with normal arteries (Galea et al., 1996; Shi X. et al., 2015; Paramel Varghese et al., 2016). IL-1β inhibition by canakinumab evidently reduces inflammation in patients with atherosclerotic disease (Choudhury et al., 2016), and reduces recurrent cardiovascular events in stable patients with coronary artery disease (Ridker et al., 2017). Thus inhibition of GSDMD cleavage by Ac-FLTD-CMK may be of therapeutic benefit in ASCVD patients, and further studies in ASCVD patients need to be undertaken.

GSDMD is cleaved by inflammatory caspases (such as caspase-1) into GSDMD-N oligomerizing to form pores in membranes, thereby inducing pyroptosis (Yang et al., 2018). In this context, the deficiency of GSDMD had ameliorated vascular inflammation and reduced lesion development (Opoku et al., 2021). The same effects are induced by a lack of caspase-1 or other pyroptosis-related proteins (Kirii et al., 2003; Gage et al., 2012; Usui et al., 2012; Yin et al., 2015) in ApoE^−/−^ mice. Inhibition of GSDMD activation may therefore be a promising and potential therapeutic target for treating atherosclerosis. A few inhibitors targeting the GSDMD activation pathway and GSDMD itself have been developed in recent years; these include Z-VAD-FMK and disulfiram. However, most of them have low specificity and are associated with a high risk of side effects. For example, Z-VAD-FMK was originally developed to restrain inflammatory caspases (Slee et al., 1996; Burdette et al., 2021) and it significantly inhibits GSDMD activation and pyroptosis in macrophages (Yang et al., 2018). Notably, Z-VAD-FMK does not specifically block only the inflammatory caspases; it also suppresses apoptotic caspases (Burdette et al., 2021). The lack of distinction between caspases leads to off-target effects, rendering it inappropriate for use as a drug (Slee et al., 1996; Ekert et al., 1999; Burdette et al., 2021). Moreover, disulfiram, an inhibitor of the enzyme acetaldehyde dehydrogenase, has been proven to effectively block GSDMD activation and GSDMD-induced pore formation; it inhibits pyroptosis in cells and LPS-induced septic death in mice (Hu et al., 2020; Burdette et al., 2021). Nevertheless, it has been found to have inhibitory effects on various proteins with diverse functions (Burdette et al., 2021). Reports therefore suggest that disulfiram has a considerable number of side effects (Watson et al., 1980; Christensen et al., 1984; Berlin, 1989). Ac-FLTD-CMK has been designed based on the specific cleavage site peptide of human GSDMD and has been proven to significantly block GSDMD cleavage and pyroptosis by potently and specifically inhibiting the enzymatic activities of caspases 1, 4, 5, and 11, but not the apoptotic caspases (such as caspase-3) (Yang et al., 2018). The specificity and availability of Ac-FLTD-CMK has been demonstrated by a series of pharmacological and cellular assays (Yang et al., 2018). In addition, polypeptide drugs are commonly and safely used in clinical practice (Lau and Dunn, 2018; Danielsen et al., 2022). Thus, in our study, Z-LLSD-FMK was designed as the specific cleavage site peptide of mouse GSDMD _273_LLSD_276_, and it was found to inhibit GSDMD activation *in vivo* and *in vitro* and decrease lesion development in ApoE^−/−^ mice. In addition to targeting the upstream pathways of GSDMD activation or GSDMD itself, IL-1β has also been evaluated in clinical studies for the treatment of ASCVDs and has been proven to be effective to a certain extent (Choudhury et al., 2016; Ridker et al., 2017). However, it is a downstream effector protein of pyroptosis; GSDMD, a final executor of canonical and non-canonical inflammasome activity or pyroptosis, is required for IL-1β secretion (He et al., 2015; Shi et al., 2017). Inhibition of GSDMD activation may be a better therapeutic strategy, as the pyroptosis pathway and inflammation are inhibited earlier upstream. Ac-FLTD-CMK may therefore be a promising and potential therapeutic drug for the treatment of atherosclerosis in the future.

Collectively the results of the present study indicate that suppression of GSDMD activation by the novel mouse GSDMD polypeptide inhibitor Z-LLSD-FMK and the specific caspase-1 inhibitor Z-YVAD-FMK significantly reduces vascular inflammation and atherosclerotic lesion development in ApoE^−/−^ mice. Both of them markedly restrained GSDMD activation or pyroptosis in a BMDM model of LPS + nigericin. Our findings suggest that blocking GSDMD activation by the novel human GSDMD polypeptide inhibitor Ac-FLTD-CMK may be a novel and effective strategy for reducing atherosclerotic lesion development for the treatment of atherosclerosis.

## Data Availability

The original contributions presented in the study are included in the article, further inquiries can be directed to the corresponding authors.
